# Management outcome of epidural hematoma patients at Dessie Comprehensive Specialized Hospital: one-year prospective observational study

**DOI:** 10.3389/fsurg.2025.1381042

**Published:** 2025-11-28

**Authors:** Belachew Tegegne, Abebaw Welelaw, Mekuriaw Wuhib Shumye, Leul Mekonnen Zeru

**Affiliations:** 1 Department of Nursing, College of Medicine and Health Sciences, Injibara University, Injibara, Ethiopia; 2 Department of Surgery, School of Medicine, College of Medicine and Health Sciences, Debre Markos University, Debre Markos, Ethiopia; 3 Department of Comprehensive Nursing, School of Nursing and Midwifery, College of Medicine and Health Sciences, Wollo University, Dessie, Ethiopia

**Keywords:** factors, epidural hematoma, head injury, management outcome, Ethiopia

## Abstract

**Background:**

Epidural hematoma is a life-threatening neurosurgical emergency that requires prompt intervention. In Ethiopia, epidural hematoma is linked to a high prevalence of morbidity and mortality. Despite the high burden of traumatic brain injuries in the region, there is a lack of local data on the management outcomes of epidural hematoma in Ethiopia.

**Objective:**

To assess the management outcomes and associated factors of epidural hematoma at Dessie Comprehensive Specialized Hospital.

**Methods:**

An institution-based prospective observational study was conducted among 46 patients at Dessie Comprehensive Specialized Hospital from January 2022 to January 2023. Data were extracted from morbidity/mortality reports, hospital records, and patient cards. Data were coded, entered into EpiData version 3.1, and analyzed using SPSS version 23. Chi-square test was used to identify factors associated with outcomes of epidural hematoma.

**Results:**

Among patients, 82.6% had good recovery, 10.9% severe disability, 2.2% persistent vegetative state, and 4.3% died. Injury mechanism (*p* = 0.001), headache history (*p* = 0.028), Glasgow Coma Scale score (*p* = 0.001), aspiration (*p* = 0.001), hypotension (*p* = 0.001), elevated intracranial pressure (*p* = 0.001), pupillary signs (*p* = 0.001), body weakness (*p* = 0.001), intracranial injury manifestation (*p* < 0.001), TBI severity (*p* = 0.001), computed tomography (CT) findings (*p* = 0.001), surgical procedure type (*p* = 0.003), and intensive care unit admission (*p* < 0.001) were significantly associated with the management outcomes of epidural hematoma.

**Conclusion:**

The majority of patients experienced favorable clinical outcomes. Outcomes of epidural hematoma management significantly associations with the mechanism of injury, presence of aspiration, prior headache history, Glasgow Coma Scale score, hypotension, elevated intracranial pressure, pupillary abnormalities, focal neurological deficits, and traumatic brain injury severity. Implementing context-specific policy interventions, enhanced safety protocols, and targeted public education initiatives could substantially reduce the incidence and impact of epidural hematomas in Ethiopia.

## Introduction

Epidural hematoma (EDH) is a life-threatening neurosurgical emergency that requires prompt diagnosis and intervention to prevent severe morbidity and mortality. It is commonly caused by traumatic head injury, leading to arterial bleeding between the skull and the dura mater. Without timely surgical evacuation, increased intracranial pressure can result in brain herniation and death (1, 2).

More than 70 million people in Ethiopia are under 30 years (3). The median age is 19.5 years. Road traffic accidents are leading causes of death in Ethiopia (4). EDH is a sever complication of head trauma. Studies from hospitals in Ethiopia showed that head trauma is common among trauma admissions, EDH represents a significant proportion of traumatic intracranial hematomas (5–8).

In low-resource settings like Ethiopia, managing EDH poses significant challenges due to limited access to neurosurgical care, delayed patient presentation, inadequate imaging facilities, and a shortage of trained healthcare professionals (9, 10). Dessie Comprehensive Specialized Hospital serves a large population but may face constraints in providing optimal care for EDH patients (11).

Despite the high burden of traumatic brain injuries in the region, there is a lack of local data on the management outcomes of EDH patients at Dessie Comprehensive Specialized Hospital. Understanding the clinical characteristics, treatment approaches, and patient outcomes (such as mortality rates, postoperative complications, and functional recovery) is crucial for improving care protocols and resource allocation (12, 13).

This study aims to assess the management outcomes of EDH patients at Dessie Comprehensive Specialized Hospital, identifying factors associated with poor prognosis and gaps in current practices. The findings will provide evidence-based recommendations to enhance neurosurgical care and patient survival in similar low-resource settings (14, 15).

By investigating these issues, this study will contribute to improving emergency neurosurgical care for EDH patients in Ethiopia and comparable settings (16).

## Methods and materials

### Study area, period and design

An institution-based, prospective observational study was carried out at Dessie Comprehensive Specialized Hospital (DCSH) from January 2022 and January 2023. Dessie is located 401 kilometers away from Addis Ababa, the capital city of Ethiopia. DCSH has 866 staffs’ and 422 beds. DCSH serves over 10 million people. The department of surgery provides residency training in addition to surgical case treatment.

### Study participant

Participants in the study were all individuals with epidural hematomas.

### Inclusion and exclusion criteria

All patients with epidural hematoma were included. Unconfirmed CT scan findings, incomplete medical records, patients who had a history of coagulopathy, and patients’ on long-term anticoagulant therapy were excluded from the study.

### Variables and operational definitions

A dichotomy of good outcome and poor outcome based on GCS and neurological status was defined for our cohort. The baseline variables considered for statistical analysis included: socio-demographic factors, pattern and mechanism of injury, clinical presentation, and CT scan findings. Details of those variables as well as of the operational definitions are provided in Tables 1, 2.

**Table 1:** Baseline variables and operations definitions.

Category	Variables	Operational definitions
Sociodemiographic	Age, gender, occupation	Age in years, gender(M/F), occupation (manual/office/other)
Injury pattern	Blunt vs. penetrating	Mechanism of trauma (Blunt force vs. Sharp object)
Clinical presentation	GCS score, pupillary response	GCS at admission (3–15); pupillary reactivity(present/absent)
Clinical findings	Midline shift, Hematoma	Radiological evidence of midline shift(mm); hematoma type(SDH/EDH/Contusion

**Table 2:** Outcome dichotomy definition.

Outcome category	Definition
Good outcome	GCS ≥ 13 at discharge and no major neurological deficit(modified Rankin ≤2)
Poor outcome	GCS ≤ 13 at discharge or Significant neurological impairment(modified Rankin ≥3)

### Data collection tools and procedures

A structured data abstraction sheet was used to collect the data. The total number of patients at the time of data collection was 46. The data was gathered from hospital records, morbidity and mortality reports, and the patient's medical card.

### Data quality assurance

Training was given to supervisors and data collectors about objective of study and how to extract variables from patient cards and registration books. A pre-test was carried out to make sure the tool was consistent and applicable.

### Data processing and analysis

After being coded, the gathered data was imported into EpiData version 3.1 and then exported to SPSS version 23 for analysis. The factors associated with epidural hematoma were found using the chi-square test. Texts, tables, and figures were used to show the results.

## Results

### Socio demographic characteristics

Most patients (84.8%) were males, and the mean (± SD) age was 29.96 ± 12.19 years. Eleven patients (23.9%) were drivers (Table 3).

**Table 3:** Sociodemographic characteristics of participants (*n* = 46).

Variables	Variable categories	Frequency	Percentage
Age	<20 years	12	26.1
	20–40 years	26	56.5
	>40 years	8	17.4
Mean (±SD)	29.96 (±12.19) years		
Sex	Male	39	84.8
	Female	7	15.2
Occupation	Farmer	10	21.7
	Driver	11	23.9
	Merchant	5	10.9
	Employee	4	8.7
	Military	9	19.6
	Others^a^	7	15.2

a Students, housewife, retirement, SD, standard deviation.

### Pattern and mechanism of traumatic head injury

According to the mechanism of injury, car accidents accounted for 41.3% of trauma. Over half (56.5%) of the patients came within 24 h of the accident, and 43.5% of them had happened outdoors. Regarding consciousness, 97.8% of patients had a history of lost consciousness, and 73.9% of patients had lost consciousness for more than 30 min (Table 4).

**Table 4:** Pattern and mechanism of traumatic head injury.

Variables	Variable categories	Frequency	Percent
Mechanism of injury	Road traffic accident	19	41.3
	Falls	7	15.2
	Fighting	17	37.0
	Bullet injury	3	6.5
Time of arrival after injury	<2 h	2	4.3
	2–8 h	7	15.2
	8–24 h	11	23.9
	>24 h	26	56.5
Place of occurrence	Vehicle	8	17.4
	Pedestrians	9	19.6
	Outdoor	20	43.5
	Home	9	19.6
History of loss of consciousness	Yes	45	97.8
	No	1	2.2
Duration of consciousness	5–30 min	12	26.1
	>30 min	34	73.9
History of abnormal body moment	Yes	7	15.2
	No	39	84.8
History of headache	Yes	45	97.8
	No	1	2.2
History of vomiting	Yes	39	84.8
	No	7	15.2

### Clinical presentations

Upon admission, patients showed up in a variety of ways: two out of five (41.3%) had a GCS level of 9–13, and 71.7% had lost consciousness. More than one-third (34.8%) of patients experienced aspiration problems, and 14 (30.4%) patients had elevated intracranial pressure. More than half (58.6%) had reactive, midsized pupils, and 58.7% patients had hemiparesis. The percentages of patients with mild, moderate, and severe TBIs were 47.8%, 41.3%, and 10.9%, respectively (Table 5).

**Table 5:** Clinical presentations.

Variables	Variable categories	Frequency	Percentage
GCS level	3–4	0	0
	5–8	5	10.9
	9–13	19	41.3
	14–15	22	47.8
Loss of consciousness	Yes	33	71.7
	No	13	28.3
Having seizure	Yes	4	8.7
	No	42	91.3
Aspiration	Yes	16	34.8
	No	30	65.2
Hypotension (SBP < 90 mmHg)	Yes	3	6.5
	No	43	93.5
Increased ICP	Yes	14	30.4
	No	32	69.6
Symptoms of Increased ICP	Vomiting	2	4.3
	Decreased mentation	6	13.0
	All^a^	6	13.0
Pupillary sign	Midsized & reactive	27	58.7
	Unilaterally dilated & fixed	15	32.6
	Bilaterally dilated & fixed	4	8.7
Body weakness	No	20	43.5
	Monoparesis	4	8.7
	Hemiparesis	15	32.6
	Hemiplegia	4	8.7
	Others^b^	3	6.5
Severity of TBI	Mild TBI	22	47.8
	Moderate TBI	19	41.3
	Severe TBI	5	10.9
Associated other intracranial injury	Yes	39	84.8
	No	7	15.2
Manifestations of intracranial injury	Brain contusion	10	25.6
	Intracranial hemorrhage	7	17.9
	Skull bone fracture	22	56.4
Associated extra cranial injury	Yes	12	26.1
	No	34	73.9
Manifestations of extra cranial injury	Extremity bone fracture	7	58.3
	Chest injury	4	33.3
	Abdominal injury	1	8.3

a All: if the patient has vomiting, decreased mentation and others associate with ICP.

b Others: paraplegia and quadriplegia.

### CT scan findings

Results from CT scan showed that 54.3% of patients had parieto-temporal epidural hemorrhages. More than two-thirds (67.1%) of patients had hemorrhages larger than 1.5 cm. The distribution of hematoma laterality was as follows: bilateral involvement (10.9%), left hemisphere (31.1%), and right hemisphere (50.0%). Over half (52.2%) of patients experienced a midline shift greater than 5 mm. In 37.0% of cases, depressed skull fractures were found (Table 6).

**Table 6:** CT scan findings.

Variables	Variable category	Frequency	Percentage
Sites of EDH	Frontal	12	26.1
	Temporal	8	17.4
	Parito-temporal	25	54.3
	Occipital	1	2.2
Thickness of the hematoma	<1.5 cm	15	32.6
	>1.5 cm	31	67.1
Involvement of Hemisphere	Right	23	50.0
	Left	18	39.1
	Bilateral	5	10.9
Midline shift	No	8	17.4
	<5 mm	14	30.4
	>5 mm	24	52.2
Other CT findings	None	7	15.2
	Linear skull fracture	6	13.0
	DSF	17	37.0
	Contusion	14	30.4
	Others	2	4.3

### Management approaches

During the study period, 46 patients experienced epidural hematomas. Of these, 10 cases were treated conservatively, while 36 cases were treated surgically. Of the 36 surgically managed cases, 20 were managed by craniotomy, 16 were managed by elevation. Patients undergoing surgery were put under general anesthesia.

### Management outcome of EDH

**Figure 1:**
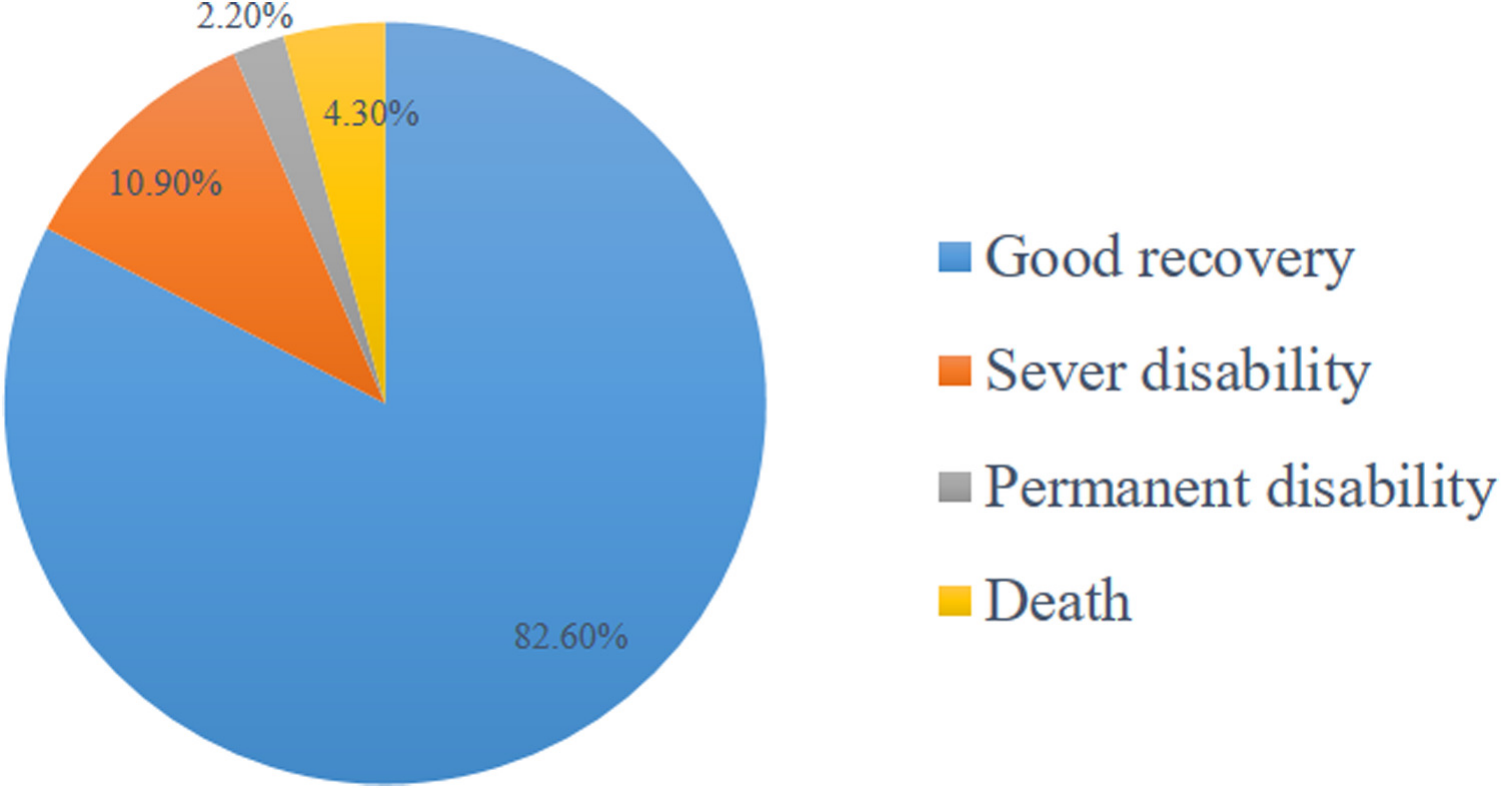
Management outcome of epidural hematoma.

The finding revealed that 38 (82.6%) recovered well, 5 (10.9%) experienced severe disability, 1 (2.2%) remained in a persistent vegetative state, and 2 (4.3%) died (Figure 1). Persistent vegetative state, death, and severe disability were considered as poor outcomes. Accordingly, the results showed that 82.6% (95% CI: 73.9–95.7) of the patients had good outcomes, while 17.4% (95% CI: 4.3–26.1) had poor outcomes.

### Factors associated with the management outcome of EDH

A chi-square (*X*²) test was used to identify the variables related to the management outcome of EDH. Mechanism of injury (*P* = 0.001), headache history (*P* = 0.028), GCS (*P* = 0.0001), aspiration (*P* = 0.0001), hypotension (*P* = 0.0001), increased intracranial pressure (*P* = 0.0001), presence of pupillary signs (*P* = 0.0001), body weakness (*P* = 0.001), severity of TBI (*P* = 0.0001), intracranial injury manifestations (*P* = 0.001), CT scan findings (*P* = 0.001), type of surgical intervention (*P* = 0.003), and ICU admission (*P* = 0.0001) were all found to be significantly correlated with the management outcome of epidural hematoma(Table 7).

**Table 7:** Factors associated with management outcome of epidural hematoma.

Variables	Variable category	Outcome		df	Chi-square *P*-value
		Good	Poor		
Age	<20 years	11	1	2	0.222
	20–40 years	22	4		
	>40 years	5	3		
Sex	Male	33	6	1	0.397
	Female	5	2		
Occupation	Farmer	8	2	5	0.689
	Driver	10	1		
	Merchant	4	1		
	Employee	4	0		
	Military	6	3		
	Others	6	1		
Mechanism of injury	Road traffic accident	15	4	3	0.001
	Falls	7	0		
	Fighting	16	1		
	Bullet injury	0	3		
Time of arrival after injury	<2 h	2	0	3	0.911
	2–8 h	6	1		
	8–24 h	9	2		
	>24 h	21	5		
Place of occurrence	Vehicle	7	1	3	0.892
	Pedestrians	7	2		
	Outdoor	16	4		
	Home	8	1		
History of loss of consciousness	Yes	37	8	1	0.68
	No	1	0		
Duration of consciousness	5–30 min	12	0	1	0.064
	>30 min	26	8		
History of abnormal body moment	Yes	5	2	1	0.397
	No	33	6		
History of Headache	Yes	38	7	1	0.028
	No	0	1		
History of Vomiting	Yes	31	8	1	0.187
	No	7	0		
Glasgow comma scale level	5–8	0	5	2	0.0001
	9–13	16	3		
	14–15	22	0		
Loss of consciousness	Yes	25	8	1	0.051
	No	13	0		
Having seizure	Yes	3	1	1	0.674
	No	35	7		
Aspiration	Yes	8	8	1	0.0001
	No	30	0		
Hypotension (SBP < 90 mmHg)	Yes	0	3	1	0.0001
	No	38	5		
Increased Intracranial pressure	Yes	6	8	1	0.0001
	No	32	0		
Pupillary sign	Midsized & reactive	27	0	2	0.0001
	Unilaterally dilated & fixed	11	4		
	Bilaterally dilated &Fixed	0	4		
Body weakness	No	20	0	4	0.001
	Mono paresis	3	1		
	Hemiparesis	8	2		
	Hemiplegia	7	2		
	Others	0	3		
Severity of Traumatic brain injury	Mild	22	0	2	0.0001
	Moderate	16	3		
	Severe	0	5		
Associated intracranial injury	Yes	31	8	1	0.187
	No	7	0		
Manifestations of intracranial injury	Brain contusion	6	4	2	0.001
	Intracranial hemorrhage	3	4		
	Skull bone fracture	22	0		
Sites of epidural hematoma	Frontal	8	4	3	0.242
	Temporal	6	2		
	Parito-temporal	23	2		
	Occipital	1	0		
Thickness of the hematoma	<1.5 cm	14	1	1	0.182
	>1.5 cm	27	4		
Involvement of Hemisphere	Right	19	4	2	0.985
	Left	15	3		
	Bilateral	4	1		
Midline shift	No	8	0	2	0.253
	<5 mm	12	2		
	>5 mm	18	6		
Other CT findings	None	7	0	4	0.001
	Linear skull fracture	6	0		
	Depressed skull fracture	17	0		
	Contusion	7	7		
	Others	1	1		
Type of surgical intervention	Craniotomy	12	8	1	0.003
	Elevation & evacuation	17	0		
Intensive care unit admission	Yes	4	10	1	0.0001
	No	30	2		

df, degree of freedom.

## Discussion

Head trauma can have a wide range of mechanisms, and the specifics of the adult and pediatric traumatic brain injuries in Ethiopia have been previously described by others (17, 18). The purpose of this study is to identify the management outcome of EDH and associated factors among patients attending Dessie Comprehensive Specialized Hospital.

In this study, 2.2% of the participants were in a vegetative state, 10.9% had a significant disability, 82.6% had fully recovered, and 4.3% had died. Overall, 17.4% (95% CI: 4.3%–26.1%) had poor outcomes, compared to 82.6% (95% CI: 73.9%–95.7%) who had good outcomes. This result is consistent with research from Addis Ababa (19) and Jimma (20), Ethiopia. The consistency with Addis Ababa and Jimma studies suggests that the observed outcomes are representative of neurological recovery patterns in Ethiopia's healthcare context.

This study's poor outcome is 17.4% (95% CI: 4.3–26.1). This is consistent with studies conducted at Amhara regional comprehensive hospitals (12.13%) (21). Both reflect Ethiopia's healthcare realities and statistical overlap confirms no major discrepancy.

On the other hand, 82.6% (95% CI: 73.9%–95.7%) of patients had good outcomes. This is in line with previous studies: Kenya (86.2%) (22), Spanish (81.5%) (23), Pakistan (92%) (24) and Italy (86%) (25). This good outcome is also supported by guidelines that state that good outcomes may be seen in 85%–90% of patients with rapid CT scans and intervention (26).

Outcomes of EDH were strongly associated with increased intracranial pressure and the severity of TBI. This is consistently good with hospitals in the Amhara region (21). Increased ICP directly worsens outcomes by causing brain herniation and Secondary ischemic injury. This is a universal medical principle, explaining why the study's findings align with global literature.

According to the mechanism of injury, traffic accidents were very common. This finding is similar to studies in Ethiopia (19, 20). Road traffic accidents dominate TBI epidemiology in Ethiopia due to poor road infrastructure (e.g., lack of guardrails, pedestrian crossings), overcrowded vehicles (e.g., minibuses, motorcycles) and weak traffic law enforcement (speeding, drunk driving).

A low GCS was significantly associated with a bad epidural hematoma prognosis. This conclusion is supported by previous studies (19, 24, 27, 28). This is because a low GCS level in a patient may be a sign of a poor prognosis. Likewise, there is a positive correlation between a bad pupillary sign and a poor epidural hematoma prognosis. This is supported by earlier research (29). This is because individuals who are nearing the end of their lives have fixed and dilated pupils. This is a sign of impending death.

Hypotension is linked to a worse prognosis in cases with epidural hematoma. This is consistent with a prior Chinese study (28). This can be explained by the fact that dehydration and bleeding happen during surgery. Bleeding may worsen and ultimately have a negative impact if their blood pressure is lower than normal.

Aspiration contributed to the unfavorable prognosis of EDH. This is consistent with previous research (30). EDH patients often have depressed consciousness which increases aspiration. Aspiration leads to pneumonia and directly compounds the primary brain injury, worsening outcomes.

Elevated intracranial pressure was strongly linked to patient mortality. This result is in line with other researches (23, 25). This is because the brain requires a constant supply of oxygen and glucose, and the cranial cavity has limited space to expand. When intracranial pressure is increased, a brain herniation occurs which results in death. In fact, a study from Prisco et al. demonstrated that high blood glucose and base excess, low mean arterial pressure, PaO2/FiO2 ratio and serum hemoglobin are all predictive of poor outcome and mortality in traumatic brain injury patients admitted to intensive care unit (31, 32).

There is a substantial correlation between the results of the CT scan and the EDH management outcome. This is supported by earlier research (33). CT is the gold standard for EDH detection due to its ability to identify to detect active bleeding, midline shift, and herniation signs—critical for surgical decision-making. Accurate diagnosis via CT directly informs life-saving interventions (e.g., craniotomy vs. conservative management).

The CT scan results showed that more than one-third (37.0%) of the patients had a depressed skull fracture; elevation and evacuation were used as treatment. This is in line with past studies carried out in Ethiopia and elsewhere (19, 21, 24, 34). In Ethiopia, road traffic accidents (dominant TBI cause) frequently involve pedestrians struck by vehicles or motorcycle crashes, generating direct focal impacts ideal for causing depressed fractures. This is also in keeping with the Brain Trauma foundation guidelines for the management of depressed skull fractures and the standard operating procedures adopted in most major trauma centers (35, 36).

In this study, analysis of patient arrival time relative to injury revealed no statistically significant correlation with EDH outcomes. However, this finding contrasts with established clinical guidelines and published medical literatures, which consistently identify early presentation following head trauma as a significant risk factor for developing EDH. This divergence may be attributed to methodological limitations within the current study, notably its limited sample size and restricted observation period, potentially obscuring the relationship observed in broader clinical practice.

### Limitation of the study

This study has various limitations including the number of missing data despite its prospective design, and the paucity of previous literature available for comparison. Nonetheless it was worth reporting our experience which has high internal validity to address such knowledge gap and contribute to the conversation regarding neurotrauma management in low income countries.

## Conclusion

The majority of patients experienced favorable clinical outcomes. Outcomes in EDH management demonstrated significant associations with the mechanism of injury, presence of aspiration, headache history, Glasgow Coma Scale score, hypotension, elevated intracranial pressure, pupillary abnormalities, focal neurological deficits, and traumatic brain injury severity.

### Recommendations

As such the following recommendations at community, hospital and policy levels can be drawn to increase the quality of care delivered to trauma patients in Ethiopia.

#### Hospital level

Hire neurosurgeons, update surgical equipment's, and prioritize timely surgical intervention.

#### Community level

Promote trauma prevention techniques like conflict avoidance, and transportation safety, as well as early presentation to hospitals.

#### Policy level

The Ethiopian Ministry of health should implement legislation, safety regulations and public awareness campaigns to reduce the prevalence of TBI.

## Data Availability

The original contributions presented in the study are included in the article/Supplementary Material, further inquiries can be directed to the corresponding author.
